# Using the integration of human resource management strategies at district level to improve workforce performance: analysis of workplan designs in three African countries

**DOI:** 10.1186/s12960-023-00838-0

**Published:** 2023-07-24

**Authors:** Tim Martineau, Wesam Mansour, Marjolein Dieleman, Patricia Akweongo, Samuel Amon, Kingsley Chikaphupha, Paul Mubiri, Joanna Raven

**Affiliations:** 1 Liverpool School of Tropical Medicine, Liverpool, United Kingdom; 2 KIT Royal Tropical Institute, Amsterdam, The Netherlands; 3 Athena Institute, VU University, Amsterdam, The Netherlands; 4 Department of Health Policy, Planning and Management, School of Public Health, College of Health Sciences, University of Ghana, Legon, Accra, Ghana; 5 Research for Equity and Community Health (REACH) Trust, Lilongwe, Malawi; 6 Makerere University School of Public Health, Kampala, Uganda

**Keywords:** Health workforce, Human resource management, District planning, Workforce performance

## Abstract

**Background:**

There is a worldwide shortage of health workers against WHO recommended staffing levels to achieve Universal Health Coverage. To improve the performance of the existing health workforce a set of integrated human resources (HR) strategies are needed to address the root causes of these shortages. The PERFORM2Scale project uses an action research approach to support district level management teams to develop appropriate workplans to address service delivery and workforce-related problems using a set of integrated human resources strategies. This paper provides evidence of the feasibility of supporting managers at district level to design appropriate integrated workplans to address these problems.

**Methods:**

The study used content analysis of documents including problem trees and 43 workplans developed by 28 district health management teams (DHMT) across three countries between 2018 and 2021 to identify how appropriate basic planning principles and the use of integrated human resource and health systems strategies were used in the design of the workplans developed. Four categories of HR strategies were used for the analysis (availability, direction, competencies, rewards and sanctions) and the relationship between HR and wider health systems strategies was also examined.

**Results:**

About half (49%) of the DHMTs selected service-delivery problems while others selected workforce performance (46%) or general management (5%) problems, yet all workplans addressed health workforce-related causes through integrated workplans. Most DHMTs used a combination of strategies for improving direction and competencies. The use of strategies to improve availability and the use of rewards and sanctions was more common amongst DHMTs in Ghana; this may be related to availability of decision-space in these areas. Other planning considerations such as link between problem and strategy, inclusion of gender and use of indicators were evident in the design of the workplans.

**Conclusions:**

The study has demonstrated that, with appropriate support using an action research approach, DHMTs are able to design workplans which include integrated HR strategies. This process will help districts to address workforce and other service delivery problems as well as improving ‘health workforce literacy' of DHMT members which will benefit the country more broadly if and when any of the team members is promoted.

**Supplementary Information:**

The online version contains supplementary material available at 10.1186/s12960-023-00838-0.

## Introduction

### General

There is a worldwide shortage of health workers, particularly in the African and Mediterranean regions, against WHO recommended staffing levels to achieve Universal Health Coverage recommended by the World Health Organization (WHO) [1]. Making the most efficient use of the available workforce through improved health workforce performance is therefore important—both the collective and individual performance of the workforce (including skills mix, levels of absence, and quality and quantity of work output). This requires a level of literacy about the health workforce—in other words “the capacity to obtain, process and understand health workforce information and services needed to make appropriate health workforce decisions” ([2], p. 2).

The aim of this document review, based on the experience of the PERFORM2Scale project, was to assess the feasibility of designing potentially effective integrated workplans for health workforce performance improvement at district level.

### Theoretical background

Improved health workforce performance management can be more easily achieved using the principles of Strategic Human Resource Management (SHRM) [3]—an important ingredient of ‘health workforce literacy’—right from the design stage. In addition to ensuring vertical integration (i.e. the human resource (HR) strategies clearly support organisational goals), SHRM advocates horizontal integration of HR strategies—or ‘bundles of strategies’ [4, 5], e.g. training supported by supervision and performance appraisal—to ensure maximum impact. Though the use of integration of HR strategies is advocated by the Global Health Workforce strategy 2030 [6] and in the recently updated guidelines for staffing rural and remote areas [7], the successful use of this approach has not been well documented in recent reports that cover health workforce strategy [1, 8, 9]. Furthermore, there is little evidence about if and how managers acquire these important skills for designing integrated HR strategies [10].

### Supporting DHMTs to develop integrated HR strategies

The practical application of the concept of horizontal integration (or ‘coherence’ [11]), of HR strategies was incorporated into a district level management strengthening initiative (MSI) used as a pilot by the PERFORM consortium (2011–15) [12] and then scaled up by the PERFORM2Scale consortium (2017–2022) [13] in Ghana, Malawi and Uganda. In each country there are District Health Management Teams (DHMT) with similar functions and operating in varying decentralised contexts [14]. The MSI, which was facilitated by project staff from each country together with health service officials, is described in more detail in Box 1.

Box 1: guidance for choosing human resource/health system strategies **The intervention**

**Fig. 1:**
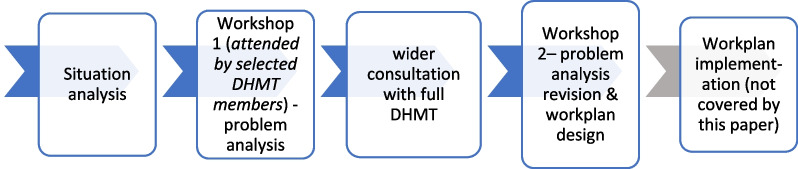
Initial stages of the MSI

The MSI is based on an action research approach [15] with groups of three District Health Management Teams (DHMTs) supported by external facilitators and peer review through meetings and short workshops [12] during which a problem of concern to the DHMT is analysed and then a set of strategies supported by appropriate activities are designed to form a workplan (see Fig. 1).

The problem selected could be related to service delivery, general management or workforce performance—what was important was that the problem was ‘owned’ by the DHMT [12]. Experience early on had shown that most workplans—regardless of the core problem being addressed—required strategies related to workforce performance. In the absence of existing guidelines on horizontal integration of HR strategies and activities, the project developed its own guidance based on multiple sources and practical experience.

**Health workforce performance strategies**

The multiple arrays of available HR strategies were grouped in the guide for convenience under four areas partially based and adapted from Vroom's expectancy theory [16], one or more of which the DHMTs selected according to the problem analysis. The first area is to examine ‘availability’ of staff for work [17]. Then, to support the performance of staff present there is a need for clear ‘direction’ relating to the work to be conducted, appropriate ‘competencies’ to complete the work effectively and appropriate ‘rewards and sanctions’ (both intrinsic and extrinsic) to encourage good performance—see Table 1.

**Table 1:** Four types of HR strategy to manage health workforce performance

HR strategy	Examples
Availability	Creating new staff posts; filling staff posts (recruitment and retention); reducing levels of staff absence
Direction	Providing information and guidance on what work staff should do (including through job descriptions; appraisal; and supervision)
Competencies	Merit-based recruitment; appraisal; and training and development
Rewards and sanctions	Strategies to influence staff behaviour and therefore their performance using financial or non-financial (including intrinsic) rewards; or sanctions (withholding benefits or taking disciplinary action)

By considering problems and relevant strategies in terms of availability, direction, competencies, and rewards and sanctions, managers were automatically guided to think in terms of ‘horizontal integration’, or ‘bundles’ of HR strategies [5]. DHMTs were guided to consider 'other health systems components' based on the WHO building blocks [18] when considering resources needed. The combined set of health workforce (referred to by the project as ‘human resource’—or ‘HR’)and health systems (or HS) strategies were referred to as ‘HR/HS strategies”. To ensure that the strategies that were eventually selected for inclusion in the workplan were relevant, participants constantly referred back to the problem analysis as they developed the workplan. Additional generic planning advice including consideration of gender and use of indicators for monitoring and evaluation (M&E) was provided.

**Fig. 2:**
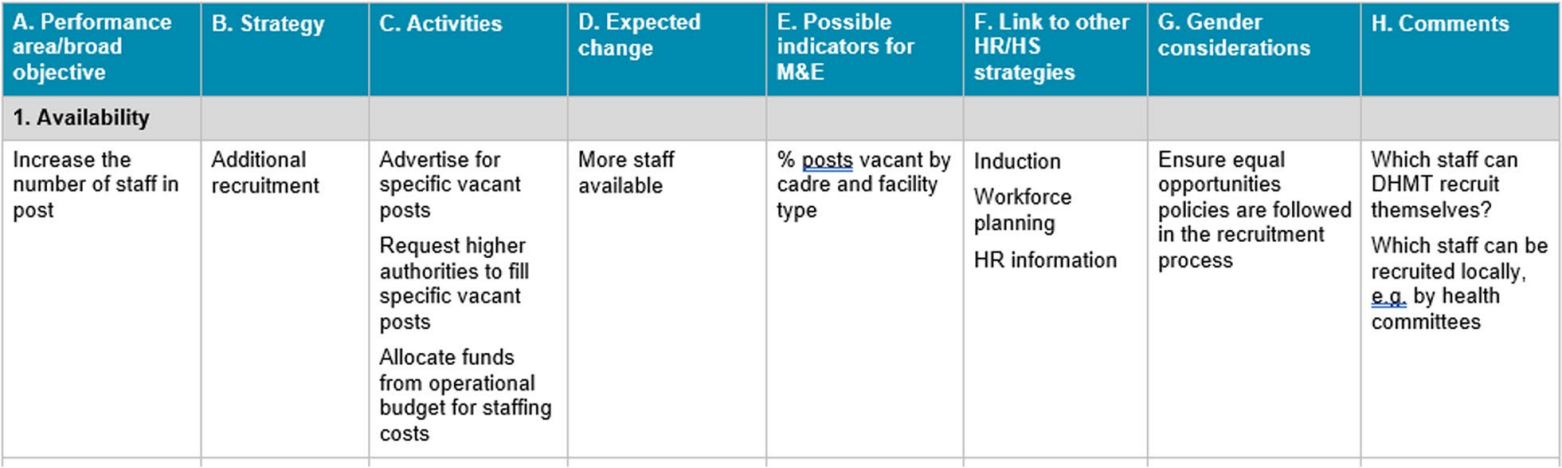
Workplan format with content for illustration

A workplan format (see Fig. 2) was provided with horizontal sections on availability, direction, competencies, and rewards and sanctions and HS and columns for inserting the different hierarchy of objectives (columns A—C)—broad objective, strategies and supporting activities [8]. Columns D and E relate to monitoring and evaluation. Column F headed “Link to/conflict with other HR/HS strategies” was included to guide managers to consider the coherence between different strategies and activities in their workplans. Column G covered gender considerations and, finally, Column H in the sample provided some prompts to help with the planning process.

A 15-page table was included in the planning guidelines for managers (see Additional file 1). The horizontal sections were divided into possible broad HR objectives—for example the area of ‘Availability’ included objectives to ‘Increase the number of staff in post’; ‘Improve distribution between rural and urban facilities’; ‘Increase number of staff present at workplace’, etc. The managers could then select objectives appropriate to their problem analysis, and suggestions were provided for sample supporting activities.

## Methods

### Research aim

The aim of the study was to assess the feasibility of designing potentially effective integrated workplans for health workforce performance improvement at district level through document review.

### Study countries and districts selected

Ghana, Malawi and Uganda were selected as countries where management has or is currently being decentralised to the district level and where consequently the district health management teams are more likely to have sufficient ‘decision space’ to address any problems identified [19]. All three countries started the implementation of the MSI initially in three districts at approximately the same time, though the scale-up to new districts and adoption of subsequent MSI cycles proceeded at a different pace in each country (see Additional file 2).

### Data collection and analysis

As part of the intervention described in Box 1 routine documentation of the programme included the collation of the problem trees and workplans developed during workshops and visits. This data collection took place between 2018 and 2021. The problem trees and the workplans were analysed using the framework provided in Table 2. Content analysis [20] was conducted for each workplan by two researchers (see Table 2) which also includes the scoring criteria. Generic planning was first reviewed in order to: categorise the type of problem statements; check the clarity of the links to the workplan; and check for any consideration of gender and inclusion of indicators. The strategies were analysed first using the four HR categories shown in Table 1. If this was not clear from the strategy column (B), the ‘activity’ column (C) (see Fig. 2) was reviewed. We have used the generic term ‘strategy’ for both levels of the ‘hierarchy of objectives’ [21] of the workplan, i.e. strategies and activities. The presence of any other health systems strategy in the workplan was then checked.

**Table 2:** Framework for reviewing the problem analysis and emergent workplans for each MSI

Strategies/content	Definitions/criteria	Scoring
*Generic planning*		
1. Type of core problem selected	Service delivery (SD); human resource (HR); general management (GM)	SD, HR or GM
2. Problem/strategy link	Clear logical link between main elements of the core problem (or lower-level cause) and emergent workplan	1 = clear link 0 = unclear or no link
3. Gender	Presence of strategies which consider gender requirements in relation to HR and/or service delivery. The guidance was to “Consider needs of men and women and how they may be affected by the strategies” and not specifically about health staff	1 = at least one example of consideration of gender 0 = no example of consideration of gender
4. Indicators	Presence of indicators for measuring change resulting from the strategy	1 = at least one example of use of indicators 0 = no example of use of indicators
*Integration of HR/HS strategies*		
5. Use of HR strategy	Number of different categories of HR strategy (availability, direction, competencies, rewards and sanctions) present in the workplan	1 = at least one example for each category 0 = no example for each category
6. Use of health systems strategy	Strategies related to information systems, finance, governance, service delivery, and medicines and health products	1 = at least one example of a health systems strategy 0 = no example of a health systems strategy

## Results

During the implementation of PERFORM2Scale, 43 workplans were developed across the three countries over two or three cycles (see Table 3). Several districts in Uganda and one in Malawi used the same problem in more than one cycle, but the workplans were modified in the second cycle—even if only slightly—and were therefore treated as different workplans. This number of workplans was developed despite the arrival of the COVID-19 pandemic in 2020 which led to some disruption to the schedule of the MSI workshops, support visits and delays to implementation of the workplans.

**Table 3:** Number of MSI workplans developed by country and district group

District group (DG)/country	#Districts	#MSI cycles	Total # workplans
Ghana			
DG1	3	2	6
DG2	3	1	3
DG3	4	1	4
Uganda			
DG1	3	3	9
DG2	3	2	6
DG3	3	1	3
Malawi			
DG1	3	2	6
DG2	3	1	3
DG3	3	1	3
Totals	28	14	43

District Group (DG) is composed of three neighbouring DHMTs. For more details see Additional file 3. In DG3 in Ghana 2 workplans were developed (one for each subdistrict) in Atiwa district

The findings represent the decisions made by the DHMTs related to the problem analysis and subsequent design of a relevant workplan and the use of the guidance in the planning table for all 43 MSI cycles (see Table 2). Some examples, where relevant, are presented for illustration.

### Type of core problem selected

Out of the 43 MSI cycles there were 21 (49%) problem statements which are based on ‘service delivery’ (SD) problems, e.g. ‘low case detection of neglected tropical disease (Yaws)’; 20 (46%) based on ‘human resource’ (HR) problems, e.g. ‘high-level absenteeism among health workers’; and two (5%) based on general management (GM) problems. The two GM problems—both from Malawi—included ‘Late data entry to DHS2 (the health information system)’ and ‘departmental heads do not compile and submit descriptive reports’.

Whereas the overall split between SD and HR problems is even, when disaggregated by country there is a clear difference between Ghana where all 13 (100%) problems were framed as relating to service delivery and between Malawi where 75% of the problems (9/12) were framed as relating to HR. In Uganda, there was a more even mix with 11 of the 18 (61%) problems being related to HR. Some switching by the DHMT after the first MSI cycle from SD to HR related problems in subsequent cycles (but not the other way around) was observed in Uganda. Some districts continued with the same problem (e.g. low tuberculosis cure rate in Cycles 1 and 2 in Luwero, Uganda; and Ntchisi and Salima districts in Malawi both worked on improving staff appraisal in both Cycles 1 and 2), though with improved workplans as the problem was not fully addressed in the first cycle. In Cycle 2, Yilo Krobo district (Ghana) continued working on the problem selected for Cycle 1 (Yaws case detection), but added other Neglected Tropical Diseases (NTDs) (leprosy and Buruli ulcer).

### Clear link between core problem and workplans

There was a clear logical link between the core problem selected and the workplan eventually produced in all 43 MSI cycles, though not all factors identified as contributing to the problem were covered by the workplan. Some of the problem areas could not be addressed within the constraints of the MSI cycle (resources, time or authority) or may not have been high priority.

### Consideration of gender

All but three (7%) workplans contained at least one reference to gender. This was mainly in relation to health staff and it was often simply noted that equal opportunity policies would be followed. However, there were some more specific factors noted such as the challenges for women using motorcycles for fieldwork in Luwero district, Uganda (Cycle 1). Nakaseke district (Uganda, Cycle 1) DHMT included strategies to improve fairness in absence management and to better disaggregate absence data by gender. Gender was also considered in service delivery elements of some plans such as differently tailored messages regarding antenatal care (ANC) attendance for males and females and the involvement of men in ANC services in Suhum district in Ghana in Cycle 1.

### Presence of indicators in workplans

Almost all (42/43–98%) workplans included at least one indicator for monitoring and evaluation and many included an indicator for each strategy in the workplan. One example from Fantekwa district, Ghana (Cycle 1) demonstrated clear strategic thinking in relation to improving staff retention. It had an activity of ‘Identify and implement both financial and non-financial incentive packages that can be contained in the District Health Authority annual budget’ for which the expected change was ‘staff motivated to accept postings in rural areas’ (Column D in the workplan—see Fig. 2) and the indicator was ‘number of vacancies in selected facilities filled with staff’.

### Use of HR strategy

All workplans—regardless of problem type (SD, HR or MD)—included one or more types of HR strategy (availability, direction, competencies and rewards/sanctions). In a few cases (6 in Ghana, 3 in Uganda and 2 in Malawi) it was necessary to review the workplans at activity level to make the categorisation. For example, Yilo DHMT (Ghana, Cycle 2) had included the activity of ‘Institute rewards for well-performing staff’ (categorised as ‘Rewards/Sanctions’) to support the broader strategy of ‘Use of health workers to search for NTD cases’ in order to address the problem of ‘Low NTDs case detection’.

On average nearly three out of the four categories of HR strategy/activity were included in the workplans (see Table 4 below), with a minimum of 2 HR categories in 15 of DHMT workplans and a maximum of 4 HR categories in 10 DHMT workplans. The inclusion of strategies related to ‘direction’ (42–97%) and ‘competencies’ (39–93%) was common in all intervention districts in the three study countries. The use of strategies related to ‘availability’ was common in Ghana at 92%; less common in Uganda at 39% and only 8% in Malawi. The use of strategies related to ‘rewards/sanctions’ was common in Ghana (85%) and less in Malawi and Uganda (both at 50%).

**Table 4:** Use of HR strategies by category and average per workplan

Categories of HR strategies	Availability (%)	Direction (%)	Competencies (%)	Reward/sanction (incentives) (%)	Av #HR categories per workplan (%)
Ghana	12/13 (92)	13/13 (100)	13/13 100)	11/13 (85)	3.77/4 (94)
Malawi	1/12 (8)	11/12 (92)	10/12 (83)	6/12 (50)	2.33/4 (58)
Uganda	7/18 (39)	18/18 (100)	17/18 (94)	9/18 (50)	2.83/4 (71)
Totals/av	20/43 (47)	42/43 (97)	39/43 (93)	20/43 (60)	2.98/4 (75)

An example of a multi-strategy workplan (including all four types of HR strategy) is found in Fantekwa District in Ghana for Cycle 1. The DHMT identified low out-patient department (OPD) attendance as the problem. Based on their problem analysis, they identified multiple strategies to include in their workplan which covered availability (lobbying for more enrolled nurses and improving retention through offering study leave and reposting staff to urban areas after serving two years in rural area; and improving attendance through regular supervision); improving direction through job description orientation and staff appraisal; improving competencies in “customer care”; and an award for the best performing staff member (reward).

In addition to the mix of categories of HR strategy, there were often multiple strategies within one category. For example, the workplan of Bunyangabu District (Uganda, Cycle 2) aimed to reduce malaria positivity rates through two complementary strategy/activities to improve ‘direction’: 1) ensuring that facility in-charges included malaria management in schedules of duties and performance plans of health workers; and 2) supervising and mentoring Village Health Teams (VHT) with a focus on malaria prevention). Two complementary strategies were also used to improve the ‘competencies’ of District Health Teams through the use of key malaria prevention guidelines and the provision of training for health assistants on key messages for sensitising VHTs on malaria prevention.

### Use of health systems strategy

Most workplans (32/43—74%) included at least one HS strategy that complemented the HR strategy (see Table 5). For example, Fantekwa’s (Ghana) Cycle 1 workplan to increase OPD attendance (described above) includes the strengthening of community engagement (classed as an HS strategy) to increase demand. Salima DHMT (Malawi, Cycle 1) complemented several HR strategies (including clearer direction and on-job training) to improve supervision with the provision of mobile phones and better transport.

**Table 5:** Number of workplans including HS strategies by study country

Inclusion of HS strategy	HS strategies (%)
Ghana	12/13 (92)
Malawi	9/12 (75)
Uganda	11/18 (61)
Total/average	32/43 (74)

Overall, Ghana and Malawi MSI workplan had more HS strategies (12/13 and 9/12, respectively) while Uganda MSI workplans had a more even mix of both HR and HS strategies. Combining HR and HS strategies does make the workplan more complex, with Fantekwa DHMT’s workplan (Ghana, Cycle 2) including 14 different strategies, but most workplans were less ambitious.

## Discussion

All DHMTs in each study country already had the competencies for making annual workplans, so it is reassuring that some of the basic elements of planning were evident in nearly all of the MSI workplans: the clear link between problem and workplans; some consideration of gender; and the use of some indicators for monitoring and evaluation of workplans.

Whereas the original PERFORM project was based on the premise that through better management of the workforce greater efficiencies would be achieved thus helping to address the global problem of workforce shortage [12], an important principle of action research (and, similarly, action learning), is that participants should have ownership of the problems they choose to address [12, 22–24]. For this reason, DHMTs were not necessarily required to address problems directly related to health workforce, though as mentioned above earlier experience indicated that regardless of the core problem being addressed the workplans required strategies related to workforce performance. It was unsurprising that about half of the problems selected related directly a service delivery, as the targets given by ministries of health to DHMTs are generally related to service delivery, such as immunisation coverage rates or tuberculosis cure rate.

Regardless of the type of problem selected, HR strategies were included in all workplans. This indicates that by carrying out a root cause analysis, DHMTs recognise that improving the management of the workforce is necessarily part of the solution to any problem they selected. This reinforces the assertion that HR is a central component of the health system [18, 25]. It is interesting to note that the DHMTs did not necessarily include HS strategies in the workplans to address workforce performance—7 HR problems in Uganda and 3 in Malawi included no HS strategies.

Having recognised the importance of including HR strategies, the DHMTs did not rely on single HR strategies such as training or supervision in their workplans to address the workforce-related problems. The DHMTs—particularly in Ghana—included a range of categories of HR strategies in their workplans which is a clear demonstration of the SHRM concept of ‘horizontal integration’, or ‘bundles’ of HR strategies—whether intended or not. The highest use of the four categories was ‘direction’ and ‘competence’. Strategies related to ‘availability’ and ‘reward/sanction’ are less likely to fall within the ‘decision space’ of the DHMTs [26–28] as these probably require external resources which DHMTs in Malawi and Uganda may have found more challenging. Although a full analysis of all strategies in the workplans was beyond the scope of this study, examples of multiple strategies within category were also identified. This further demonstrated that the DHMTs had acquired the skills from the MSI process to develop "bundles" of linked and coordinated SHRM interventions. The integration of strategies both between and within the four categories of HR strategy ‘will be more likely to achieve sustained improvements in organisational performance than single or uncoordinated interventions’ [﻿4, p7]. However, the quality of the ‘bundles’ is more important than the quantity of strategies included [29] and Marchal and Kegels suggest that it is more important that the strategies are complementary and appropriate to need [30].

The evidence presented in this paper has demonstrated that it is possible for DHMTs to design workplans with integrated HR strategies in line with SHRM thinking using the MSI approach described. As shown at district level, improving health workforce performance using integrated HR strategies is just as important for solving wider service delivery problems. This is equally true at higher levels of the health system. This 'health workforce literacy' [31] will also help DHMT members who may get promoted whether they take higher level posts in HR management or other broader areas of management to have more impact on health workforce performance improvement. The SHRM concept of ‘horizontal integration’ strategies needs to be incorporated into health management related training to support the design and implementation of bundles of HR strategies. This concept is implicit in WHO’s recently published Human Resources for Health leadership and management course materials [32], but needs to be taught using practical examples.

Evaluation of the implementation and impact of these workplans at district level is ongoing, but this simple, scalable, structured approach can help district managers *design* relevant and coherent workplans to address workforce-related problems to support more effective service delivery. The approach could also be useful at higher levels of the health system and an analysis of levels of horizontal integration in national plans could be included in future reviews.

### Policy implications

As DHMTs demonstrated the ability to link effective root cause analysis to problems they had prioritised and linking this analysis to strategy development, this kind of approach should be encouraged to improve service delivery in general at district level.

DHMTs also demonstrated the ability to develop quite complex workplans including quite a wide range of integrated HR and HS strategies. Given that all problems identified, even the service delivery ones, required HR strategies, more emphasis is needed in developing health workforce literacy at this level.

The process of problem analysis and HR strategy development following SHRM principles is also needed at higher levels of the health system. A similar approach could be used at higher levels of the health system resulting in both the development of more relevant and effective HR strategies as well as broadening health workforce literacy within the system.

### Strengths and limitations

The strength of this study is that it presents a new way of analysing the degree of integration, following the SHRM approach, in the design of workplans for improving performance management across multiple districts in three countries and the range of strategies used. This approach could also be used for different areas of the health system.

As the study was based on a document review, several limitations of the study include: the lack of data on the interconnection between strategies in the workplans; on the learning by district managers either during the workplan development process or during the implementation period; and the impact of the workplans on workforce performance and service delivery.

### Future research

In future more details could be collected on the interconnection between the different strategies in the workplans, the development of DHMTs’ planning skills as they go through multiple MSI cycles and the results of the implementation of the workplans could offer more knowledge about the effectiveness of the different strategies. More detail on the quality of both the considerations of gender and the indicators would be useful. In-depth qualitative data on the DHMTs’ perceptions and experience of the guidance and facilitation process of the MSIs would benefit future users of this approach. For such a study an independent evaluation might be more appropriate.

## Conclusion

The study has shown that it is possible to give health managers in decentralised districts the necessary skills to design workplans with integrated HR strategies using an action research approach. If the workplans are implemented, this is likely to lead to improved health workforce performance at the district level, though further evaluation is needed. The process of analysing health workforce and service delivery problems using the MSI’s action research approach is likely to improve the level of ‘health workforce literacy’—and will potentially help managers at district level (and higher levels, if promoted) to have more impact on health workforce performance improvement within the health system.

## Supplementary Information

**Additional file 1.** Choosing HR/HS strategies to improve workforce performance

**Additional file 2.** Locations of participating districts by country

**Additional file 3.** Analysis of district workplans strategies

## Data Availability

The data that support the findings of this study are available from the corresponding author, Wesam Mansour, upon reasonable request.

## Abbreviations

ANC: Antenatal care
DG: District group
DHMT: District health management team
DHS2: District health information system
GM: General management
HR: Human resource
HS: Health systems
M&E: Monitoring and evaluation
MSI: Management strengthening initiative
NTD: Neglected tropical diseases
OPD: Out-patient department
SD: Service delivery
SHRM: Strategic Human Resource Management
WHO: World Health Organization
