# Supplementary material for: Using the integration of human resource management strategies at district level to improve workforce performance: analysis of workplan designs in three African countries
Source: Hum Resour Health. 2023 Jul 24;21:57. doi: 10.1186/s12960-023-00838-0 (PMC10367416; doi:10.1186/s12960-023-00838-0)
Supplement: Supplementary file 1 — Additional file 1. Choosing HR/HS strategies to improve workforce performance [file 12960_2023_838_MOESM1_ESM.pdf]

Annex 1: Choosing strategies table

Please note: this table contains ideas for consideration, not the definitive answers to your questions

A. Performance area/broad objective	B. Strategy	C. Sample activities	D. Expected change	E. Possible indicators for M&E	F. Link to other HR/HS strategies	G. Gender considerations	H. Comments
**1. Availability**							
Increase the number of staff in post	Additional recruitment	Advertise for specific vacant posts<br>Request higher authorities to fill specific vacant posts<br>Allocate funds from operational budget for staffing costs	More staff available	% posts vacant by cadre and facility type	Induction<br>Workforce planning<br>HR information	Ensure equal opportunities policies are followed in the recruitment process	Which staff can DHMT recruit themselves?<br>Which staff can be recruited locally, e.g. by health committees
	Attraction incentives	Funding initial training with bonding<br>Offer post graduate training after certain period of service<br>Offer support for spouse and children (housing, school fees-boarding school)	More applications for jobs	# applications/ post by cadre	Workforce planning	Consider different needs of women and men (e.g. women may have more difficulty in leaving home to attend training)	Check whether the main problem is attraction or retention; often confused

A. Performance area/broad objective	B. Strategy	C. Sample activities	D. Expected change	E. Possible indicators for M&E	F. Link to other HR/HS strategies	G. Gender considerations	H. Comments
	Retention incentives	Develop career opportunities within the district<br>Sponsoring further training<br>Job redesign to increase job satisfaction[x]<br>See also attraction incentives	Fewer staff leaving (or getting transfers from) the district	Staff turnover rate	Workforce planning	Consider different needs of women and men<br>Equal opportunities for career opportunities	Check whether the main problem is attraction or retention; often confused
	Use of volunteers/ non-formal health workers	Identify tasks that could be done by volunteers<br>Develop scheme for recruiting/ training/ supporting/ compensating volunteers	Increase in volume of services provided	% coverage of specific programmes	Task shifting	Consider different needs of women and men<br>Monitor gender distribution of volunteers	Advantage of better community links; but consider the additional time needed for training and supervision
	Collaboration with other health service providers	Contracting out selected services	Improved coverage<br>Possibly improved quality	% coverage of specific programmes<br>Level of patient satisfaction	Service delivery (relates to the way that services are delivered)	Consider any gender implications	Check national policy; additional work to manage contract
Improve distribution between rural and urban facilities	Attraction incentives for rural areas only	Funding initial training with bonding	More staff available in rural areas	% posts vacant by cadre in rural districts	Workforce planning	Consider different needs of women and men (e.g. women may need	Training takes staff away from the workplace;

A. Performance area/broad objective	B. Strategy	C. Sample activities	D. Expected change	E. Possible indicators for M&E	F. Link to other HR/HS strategies	G. Gender considerations	H. Comments
		Offer post graduate training after certain period of service<br>Offer support for spouse and children (housing, school fees-boarding school)<br>Engage community e.g. through local health committee in provision of housing and other amenities		% trainees who complete the bond		more support e.g. security in rural areas; also consider access to training)<br>Monitor gender distribution of staff across urban/rural areas	may need to stagger training<br>Need to agree how “rural” is defined; there may be general public sector categorisations
	Recruit health workers from rural background	State preference for candidates from rural areas when advertising vacancies<br>Give preference to candidates from rural areas at time of recruitment	Improved staff retention	Staff turnover rate disaggregated by rural/urban background	Workforce planning<br>Information systems<br>Recruitment and selection	Monitor gender distribution of staff across urban/rural areas	Dependent on flexibility of recruitment policy
	Retention incentives for rural areas	Identify financial and/or nonfinancial incentives that can be funded from	More staff available to provide service in understaffed areas	% posts vacant by cadre in rural districts<br>% trainees who complete the bond	Less money available in operational budget for equipment and	Consider different needs of women and men	Note that ‘non-financial incentives still cost money

A. Performance area/broad objective	B. Strategy	C. Sample activities	D. Expected change	E. Possible indicators for M&E	F. Link to other HR/HS strategies	G. Gender considerations	H. Comments
		the district budget[xi]<br>Some attraction incentives may also be suitable			supplies for staff to work with		
	Use temporary staffing measures	Staff rotation for 2-3 months with additional allowances<br>Outreach services to cover specialist skills	More staff available to provide service in understaffed areas	# days/year facilities are staffed	Will probably create absences in other facilities	Consider different needs of women and men (e.g. women may need more support e.g. security in rural areas; also consider access to training)	
Increase number of staff present at workplace	Attendance monitoring	Use attendance register at facilities<br>Spot check on attendance register<br>Visible presence of facility manager at start of working day<br>Involving local health committees in attendance monitoring<br>Use data with staff for discussion	Improved attendance	# working days lost	Reduce unnecessary training courses and workshops<br>Complement strategies for increasing numbers of staff	Need to be sensitive to gender needs and roles outside the workplace i.e. are women more likely to struggle to attend given their greater domestic responsibilities?	It may be necessary to address this with incremental steps<br>If possible, the data should be made public: first amongst staff; then possibly with the local health committee. If 'secret', it will be difficult to build trust<br>Important to show that data are used

A. Performance area/broad objective	B. Strategy	C. Sample activities	D. Expected change	E. Possible indicators for M&E	F. Link to other HR/HS strategies	G. Gender considerations	H. Comments
							and that there are implications
	Rewarding good attendance	Develop simple rewards e.g. best attendance for the month award	Improved attendance; reduced	# working days lost	Reduce unnecessary training courses and workshops<br>Complement strategies for increasing numbers of staff	Consider any gender implications	
	Monitoring of dual working to understand possible reasons for absence	Collect data on government staff working in additional jobs for non-government employers (using formal or informal means)	Better information on possible cause of absence<br>No. of HW currently employed at more than one location/Total no of HW	Data collected and reviewed by DHMT on a regular basis	Reduce unnecessary training courses and workshops<br>Complement strategies for increasing numbers of staff	Monitor gender differences	This will not solve the problem of absence, but may help understanding of the causes; may be difficult to collect the data as staff
	Reward team work	Give prizes to well-performing teams	Greater collaboration of staff with each other	Prizes given on a regular basis	Reduced staff absence (need to support the team effort)	Consider gender dynamics, i.e. are teams promoting women's roles and showcasing good power-sharing approaches?	The criteria for measuring team performance must be clear and staff must trust the process for this strategy to be successful<br>Management need to be able to support and

A. Performance area/broad objective	B. Strategy	C. Sample activities	D. Expected change	E. Possible indicators for M&E	F. Link to other HR/HS strategies	G. Gender considerations	H. Comments
							develop teamwork, and to deal with conflict, and be gender/ethnic etc sensitive
Develop support systems for improving staff availability	Operational HR information system used for decision-making	Ensure regular returns from facilities<br>Establish simple database using Excel or Access<br>Schedule the production of simple reports for review at DHMT meetings	Managers know how many of what cadres needed and where<br>Understanding of staffing dynamics (e.g. age profile, retention rate)	# HR data requests by DHMT for planning and management tasks	Workforce planning<br>Information systems	Consider gender sensitivity of information system	A national information system may exist, but data is not used by DHMT
	Regular workforce planning	Develop planning schedule to link with budget planning<br>Request support from higher level HR units	Managers can plan for additional recruitment	Annual update of workforce plan		Consider any gender implications and the opportunity to address any gender imbalances	This might be included in the process of national workforce planning
**2. Direction**							

A. Performance area/broad objective	B. Strategy	C. Sample activities	D. Expected change	E. Possible indicators for M&E	F. Link to other HR/HS strategies	G. Gender considerations	H. Comments
Improve staff understanding of general work of the institution/ facility; and provide feedback on performance	Ensure staff have updated job descriptions (JD)	Develop new JDs (if none)<br>Update if JDs exist in line with specific health service package to be delivered at each level<br>Pilot the process to understand what work is involved and what difficulties might be encountered	Staff know what tasks they should perform	% staff with recently updated job descriptions	Competencies<br>Use of the work plan	Consider any gender implications	Check what authority is needed to change job descriptions; it might be possible to make minor adjustments.
	Induction/ orientation of new staff	Develop basic induction checklist<br>Brief managers in induction process<br>Assigning mentor to new staff	Staff know what tasks they should perform and know routine procedures[xii]	% staff employed in past 3 months who received a basic induction	Increased recruitment	Consider positive action approaches related to gender	Will improve productivity is staff can quickly staff working effectively
	Regular open appraisal	Develop simple process and form or adapt from existing materials<br>Re-instate existing lapsed appraisal system<br>Brief managers and staff on	Staff get feedback on performance and support	% staff appraised in past 12 months	Linked to use of updated job descriptions	Consider any gender implications	The confidential review system (ACR) has generally been discredited; the system needs to be open for staff to receive useful feedback

A. Performance area/broad objective	B. Strategy	C. Sample activities	D. Expected change	E. Possible indicators for M&E	F. Link to other HR/HS strategies	G. Gender considerations	H. Comments
		procedures and advocate benefits					
	Regular supportive supervision	Develop regular supervision schedule<br>Training supervisors in effective supportive supervision<br>Develop/adapt existing supervision tool and set of guiding principles<br>Involve community-based organisations in basic supervision	Staff get feedback on performance and support	% staff supervised in past 3 months	Link to skills development<br>Link to job descriptions	Consider any gender implications	Remote staff feel neglected without supervisory visits; however, the quality of the supervision is more important than the quantity
Improve understanding of specific daily/weekly work; and provide feedback on performance	Use of workplans	Ensure staff are provided with regular daily/weekly work plans<br>Share facility work plans and targets so staff know what needs to be done/achieved	Staff have a clear understanding of their work and can prioritise activities	% staff with minimum of weekly workplans	Link to job descriptions	Consider any gender implications	The work plans should be based on wider and longer-term plans for the district, as well as including emerging priorities where necessary

A. Performance area/broad objective	B. Strategy	C. Sample activities	D. Expected change	E. Possible indicators for M&E	F. Link to other HR/HS strategies	G. Gender considerations	H. Comments
	Use of team meetings	Ensure DHMT acts as role model for team meetings<br>Encourage facility/programme managers to establish regular team meetings for planning and reviewing progress against plans	Staff have a clear understanding of their work, can prioritise activities and improve their performance	% facilities/ programmes holding team meeting in past month	Link to work plans	Consider gender dynamics of team interactions	Team meetings may be difficult to arrange when everyone is busy, but if done well it is time well spent
**3. Competencies**							
Ensuring appropriate competencies available to carry out the work	Introduce or strengthen merit-based recruitment	Use person specification based on updated job description for selection process<br>Use tests in selection process<br>Introduce more transparent selection processes	Better skilled staff	% new staff with skills that match needs of job description	Link to increase in recruitment	Ensure equal opportunities policies are followed in the recruitment process	This process may be managed by local government, so the DHMT may only be able to influence the process
	Improving skills mix	Decisions in recruitment to align skills of new staff needed to	More appropriate skills available in the workplace	No. of physicians, nurses, and midwives (or other categories of	Link to task shifting	Consider positive action approaches related to gender	

A. Performance area/broad objective	B. Strategy	C. Sample activities	D. Expected change	E. Possible indicators for M&E	F. Link to other HR/HS strategies	G. Gender considerations	H. Comments
		deliver health services at different levels		health service providers)/Total no. of health workers			
	Task shifting	Analysis of opportunities for task shifting within team (or beyond team – including community volunteers)<br>Consult staff affected by decisions on task shifting<br>Implement task shifting plan	Better use of staff with scarce (more specialist skills)	Will depend on task shifting plan	Training and development (for taking on new skills)<br>Workforce planning	Consider positive action approaches related to gender	See WHO guidelines of task shifting for HIV/AIDS and Maternal and Newborn Health[xiii]<br>Prepare for opposition by some staff groups; develop a plan for managing the change<br>Check current scopes of practice
	Introduce regular appraisal	Competencies audit included in appraisal process<br>Train appraisers to use competencies audit	Training and development needs for individual staff identified	% staff who have undergone a competency audit	Training and development	Consider any gender implications	Staff may be anxious about their first appraisal, so prepare well
	Training and development	Competencies audit/ training needs assessments at	Staff have more appropriate competencies	# staff with adequate competencies for the job	Reduction of absence (if off-site training is used)	Consider positive action approaches related to gender	Check for other causes of performance problems before

A. Performance area/broad objective	B. Strategy	C. Sample activities	D. Expected change	E. Possible indicators for M&E	F. Link to other HR/HS strategies	G. Gender considerations	H. Comments
		individual and at team level<br><br>Identify learning opportunities in addition to formal training<br><br>Provide internet and computer access for distance learning					choosing training as the solution<br><br>Assure transparency in who is getting which training and why
**4. Rewards and sanctions**							
**Reward good performance**	Introduce team incentives	Identify behaviour to be influenced<br><br>Identify rewards that could be given<br><br>Develop system<br><br>Ensure robust system of monitoring in place	Staff perceive direct link between incentives and good performance<br><br>Improved staff performance	Incentive system introduced<br><br>Specific performance indicators related to areas of service delivery could be added	Direction (ensure staff know what they are supposed to do) – job description, work plan, etc	Consider positive action approaches related to gender	This avoids individualistic approach

A. Performance area/broad objective	B. Strategy	C. Sample activities	D. Expected change	E. Possible indicators for M&E	F. Link to other HR/HS strategies	G. Gender considerations	H. Comments
	Introduce individual incentives	Identify behaviour to be influenced<br>Identify rewards that could be given<br>Develop system<br>Orient staff on why the system is being introduced and how it will affect them<br>Ensure robust system of monitoring in place	Staff perceive direct link between incentives and good performance<br>Improved staff performance	Incentive system introduced<br>Specific performance indicators related to areas of service delivery could be added	May be detrimental to team work	Consider any gender implications	Be careful to prevent incentives as being seen as a "right"; otherwise this will end up being a general pay rise with no improvement in performance.
	Give staff additional responsibility	Expand job description	Improved job satisfaction	Level of job satisfaction (from staff survey)	Job description	Consider positive action approaches related to gender	Take care not to raise expectations of extra pay if it cannot be provided
	Transfer staff to more desirable posting	Include performance in transfer criteria	Staff motivated by possibility of being transferred to more desirable location	# transfers of staff meeting positive performance criteria	Avoid negative impact on strategies to address maldistribution	Consider positive action approaches related to gender	Use with care and ensure transparency of the system so it is perceived as fair
**Manage poor performance**	Issue verbal and written warnings	Use personnel guidelines or	More staff follow rules and regulations	# warnings given	Induction<br>Supervision<br>Appraisal	Consider any gender implications	If used in a timely way, this will reduce the need for more drastic

A. Performance area/broad objective	B. Strategy	C. Sample activities	D. Expected change	E. Possible indicators for M&E	F. Link to other HR/HS strategies	G. Gender considerations	H. Comments
		develop if not available<br>Analyse factors to identify root cause	Improved staff behaviour				sanctions e.g. withholding pay or dismissal
	Withhold pay	Ensure staff know what performance is expected<br>Ensure staff are aware this sanction might be used	More staff follow rules and regulations<br>Improved staff behaviour	# times pay withheld	Use of verbal and written warnings<br>Payroll	Consider any gender implications	May be an option for contract staff
	Transfer staff to less desirable posting	Include performance in transfer criteria	More staff follow rules and regulations<br>Improved staff behaviour	# transfers of staff meeting negative performance criteria	Avoid negative impact on strategies to address maldistribution	Consider any gender implications	Use with care and ensure transparency of the system so it is perceived as fair
	Dismiss or recommend staff for dismissal	Orient managers and supervisors on use of dismissal procedures<br>Ensure staff are aware this sanction might be used	More staff follow rules and regulations<br>Improved staff behaviour	# persons dismissed	Use of verbal and written warnings<br>Withholding pay<br>Induction<br>Recruitment	Consider any gender implications, i.e. has the member of staff been discriminated against based on gender?	Use only as last resort, especially if there are already staff shortages

A. Performance area/broad objective	B. Strategy	C. Sample activities	D. Expected change	E. Possible indicators for M&E	F. Link to other HR/HS strategies	G. Gender considerations	H. Comments
**5. Health systems**							Select activities within the control of the DHMT or lobby higher levels
**Create a decent and supportive working environment**	Ensure equipment, drugs and supplies are available	Ensure requisition/ ordering systems functioning<br>Ensure regular maintenance of equipment	Staff have equipment, drugs and supplies to carry out jobs effectively<br>Increase in staff motivation and self-respect	# stockouts<br>Level of job satisfaction (from staff survey)	Improving individual and team performance		
	Infrastructure (buildings etc)	Regular maintenance<br>Infrastructure planning	Better working facilities improving productivity and staff morale	Level of job satisfaction (from staff survey)	Staff retention	Consider/monitor any gender implications, i.e. sufficient toilets available	Well-maintained buildings may also attract more clients
	Transport	Regular maintenance<br>Transport planning	Staff able to travel more often<br>More supervision visits, especially to remoter facilities	Increase in # supervision visit	Supervision<br>Service delivery (especially outreach work)	Consider/monitor any gender implications for work-related travel	
	Information systems	Use service delivery data to monitor productivity	Strategies to improve staff productivity monitored		Workforce planning<br>Improving individual and		

A. Performance area/broad objective	B. Strategy	C. Sample activities	D. Expected change	E. Possible indicators for M&E	F. Link to other HR/HS strategies	G. Gender considerations	H. Comments
					team performance		
	Finance	Ensure not too much of the operational budget is used for additional hiring	Balance between staffing costs and operational costs		Workforce planning<br>Rewards		Availability of funds may be affected by late release of the budget; contingency plans may be needed
	Governance, and accountability	Ensure transparency of HR-related systems<br>Involve staff in planning<br>Involve staff in problem solving	Staff trust HR systems<br>Increased ownership of plans		Improving individual and team performance<br>Staff retention		
	Service delivery	Rearrange delivery of services to make best use of staff<br>Modify the way services are delivered	More efficient use of existing staff		Improving individual and team performance<br>Workforce planning<br>Scheduling and sharing resources		

Endnotes

[i] Terms in bold are explained in Annex 2

[ii] There are other important areas, but these are key ones to start with.

[iii] See MSH (2009). Strengthening Human Resource Management to improve Health Outcomes – e Manager No.1. http://www.msh.org/Documents/emanager/upload/eManager_2009No1_HRM_English.pdf

[iv] Sometimes referred to as ‘extrinsic rewards’; unlike intrinsic rewards these come from outside the job itself.

[v] Examples of more ways of improving workforce performance that could be included in discussions with the DHMTs:

Improving commitment towards the aims and targets of the district

Change the technology – employing more efficient or effective ways of carrying out tasks (e.g. introduction of ultrasound scan in antenatal care and labour to improve services). This might need changes in the direction given, the competencies and the equipment and supplies.

Increase the demand for services through health promotion activities etc.

[vi] Some problems may be very unclear when you start working on them. The problem definition could be refined as the problem itself becomes clearer.

[vii] This table is available in annex 1

[viii] Copies of this table are available in electronic form either in Word or Excel

[ix] See WHO (2007). Everybody's business. Strengthening health systems to improve health outcomes. WHO's framework for action. Geneva, WHO.

[x] E.g. providing some additional responsibility

[xi] Non-financial incentives might include improved living facilities (solar panels, monthly shopping trips)

[xii] E.g. how to get supplies needed for their work; who to contact for advice

[xiii] See WHO (2007). Task Shifting: Rational Redistribution of Tasks among Health Workforce Teams. Geneva, WHO. http://www.hrhresourcecenter.org/node/1811 and Optimizing health worker roles to improve access to key maternal and newborn health interventions through task shifting http://www.optimizemnh.org/
