# Supplementary material for: Using the integration of human resource management strategies at district level to improve workforce performance: analysis of workplan designs in three African countries
Source: Hum Resour Health. 2023 Jul 24;21:57. doi: 10.1186/s12960-023-00838-0 (PMC10367416; doi:10.1186/s12960-023-00838-0)
Supplement: Supplementary file 2 — Additional file 2. Locations of participating districts by country [file 12960_2023_838_MOESM2_ESM.docx]

**Additional file 2: locations of participating districts by country**

**PERFORM2Scale districts in Ghana**


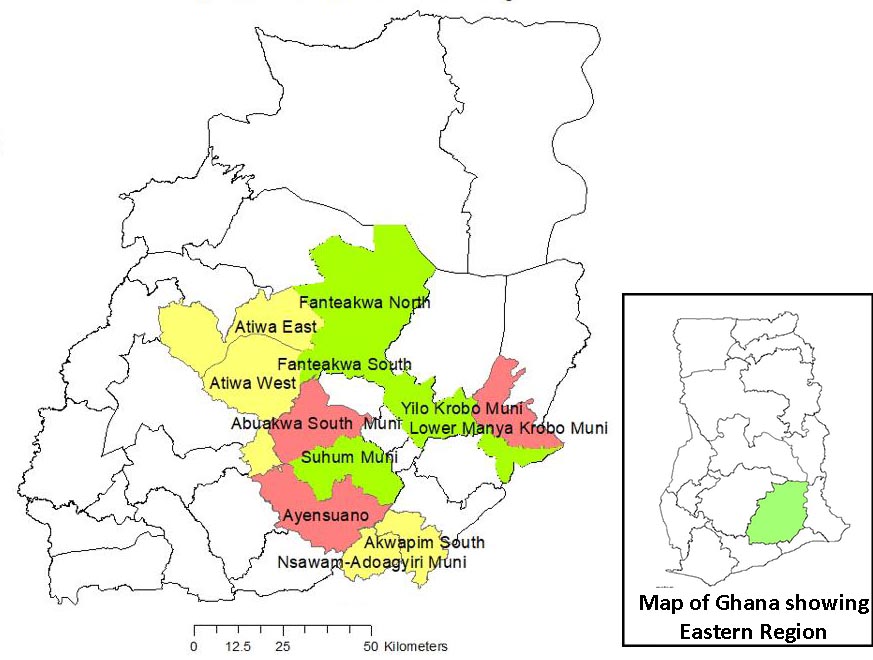


**DG1 - Fanteakwa, Yilo Krobo and Suhum**

**DG2 - Ayensuano, Lower Manya Krobo and East Akim**

**DG3 – Nsawam-Adoagyiri, Akuapim South and Atiwa**

**PERFORM2Scale districts in Malawi**


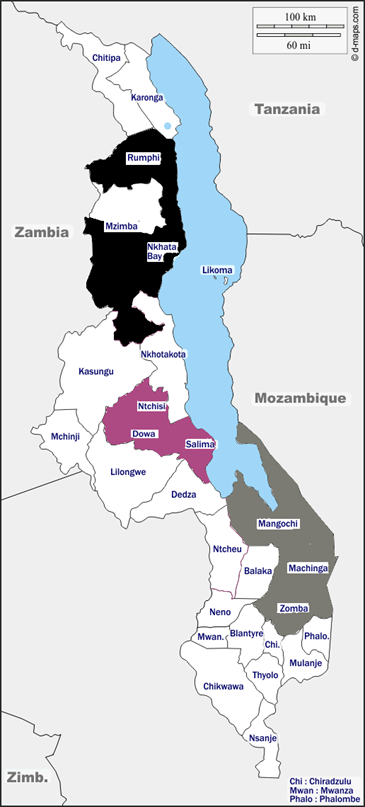


**DG1 - Dowa, Ntchisi and Salima**

**DG2 - Machinga, Mangochi and Zomba**

**DG3 - Mzimba south, Nkhata Bay and Rumphi**

**PERFORM2Scale districts in Uganda**


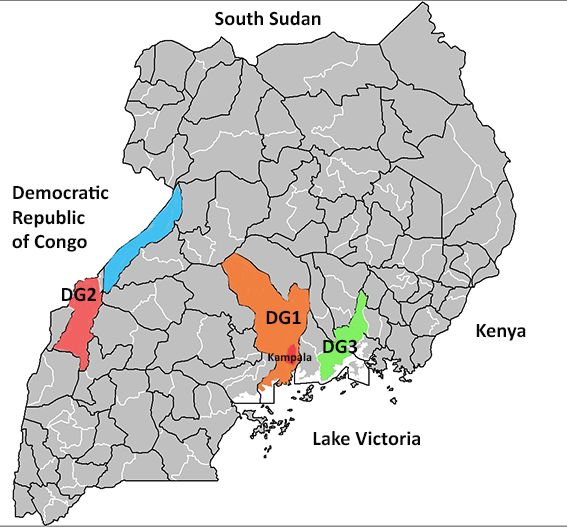


**DG1 - Luwero, Nakaseke and Wakiso**

**DG2 - Kabarole, Ntoroko and Bunyangabu**

**DG3 - Jinja, Luuka and Buikwe**
